# Subsurface fluorescence time-of-flight imaging using a large-format single-photon avalanche diode sensor for tumor depth assessment

**DOI:** 10.1117/1.JBO.29.1.016004

**Published:** 2024-01-17

**Authors:** Arthur F. Petusseau, Samuel S. Streeter, Arin Ulku, Yichen Feng, Kimberley S. Samkoe, Claudio Bruschini, Edoardo Charbon, Brian W. Pogue, Petr Bruza

**Affiliations:** a Dartmouth College, Thayer School of Engineering and Dartmouth Cancer Center, Hanover, New Hampshire, United States; b Geisel School of Medicine at Dartmouth, Department of Orthopaedics, Hanover, New Hampshire, United States; c Ecole polytechnique fédérale de Lausanne, Advanced Quantum Architecture Laboratory, Neuchâtel, Switzerland; d Geisel School of Medicine at Dartmouth, Department of Surgery, Hanover, New Hampshire, United States; e University of Wisconsin–Madison, Department of Medical Physics, Madison, Wisconsin, United States

**Keywords:** fluorescence time-of-flight, light detection and ranging, surgical guidance, single-photon avalanche diode, depth sensing

## Abstract

**Significance:**

Fluorescence guidance is used clinically by surgeons to visualize anatomical and/or physiological phenomena in the surgical field that are difficult or impossible to detect by the naked eye. Such phenomena include tissue perfusion or molecular phenotypic information about the disease being resected. Conventional fluorescence-guided surgery relies on long, microsecond scale laser pulses to excite fluorescent probes. However, this technique only provides two-dimensional information; crucial depth information, such as the location of malignancy below the tissue surface, is not provided.

**Aim:**

We developed a depth sensing imaging technique using light detection and ranging (LiDAR) time-of-flight (TOF) technology to sense the depth of target tissue while overcoming the influence of tissue optical properties and fluorescent probe concentration.

**Approach:**

The technology is based on a large-format (512×512  pixel), binary, gated, single-photon avalanche diode (SPAD) sensor with an 18 ps time-gate step, synchronized with a picosecond pulsed laser. The fast response of the sensor was developed and tested for its ability to quantify fluorescent inclusions at depth and optical properties in tissue-like phantoms through analytical model fitting of the fast temporal remission data.

**Results:**

After calibration and algorithmic extraction of the data, the SPAD LiDAR technique allowed for sub-mm resolution depth sensing of fluorescent inclusions embedded in tissue-like phantoms, up to a maximum of 5 mm in depth. The approach provides robust depth sensing even in the presence of variable tissue optical properties and separates the effects of fluorescence depth from absorption and scattering variations.

**Conclusions:**

LiDAR TOF fluorescence imaging using an SPAD camera provides both fluorescence intensity images and the temporal profile of fluorescence, which can be used to determine the depth at which the signal is emitted over a wide field of view. The proposed tool enables fluorescence imaging at a higher depth in tissue and with higher spatial precision than standard, steady-state fluorescence imaging tools, such as intensity-based near-infrared fluorescence imaging, optical coherence tomography, Raman spectroscopy, or confocal microscopy. Integration of this technique into a standard surgical tool could enable rapid, more accurate estimation of resection boundaries, thereby improving the surgeon’s efficacy and efficiency, and ultimately improving patient outcomes.

## 1 Introduction

This paper extends on previous work by Bruza et al.^1^ by providing a more comprehensive account of the methods and technical aspects involved in fluorescence depth sensing using large single-photon avalanche diode (SPAD) cameras. Additionally, this paper elaborates on the derivation of calibration curves, a crucial aspect that received limited coverage previously. A thorough review of the technique’s limitations and an introduction to quantitative measurements to support its performance are also provided. Finally, this paper proposes potential improvements to the technique and potential applications in clinical scenarios, providing valuable insights for further research and practical implementation.

Surgical resection remains a crucial approach for cancer treatment; a vast majority of breast, colorectal, lung, and bladder cancer patients undergo surgical resections as a part of standard-of-care.^2^ Although preoperative imaging has advanced significantly, the success of surgery largely depends on the surgeon’s ability to locate the pathology using conventional white light visualization and palpation.^3^^,^^4^ In the past three decades, fluorescence-guided surgery (FGS) has emerged as a promising technique for defining tumor locations and margins intraoperatively. Intraoperative visualization of tumors using FGS has the potential not only to achieve complete resections but also to improve patient safety by reducing unnecessary damage to normal tissue,^5^^–^^8^ leading to shorter operative times and a decreased likelihood of needing additional surgery. Recent developments in FGS have led to the introduction of several new imaging probes,^9^^–^^12^ each with unique spectral characteristics, tissue and/or biomarker targets, and pharmacokinetic properties, complementing existing probes and providing a growing repertoire of useful tools for FGS. Although conventional FGS techniques are useful, they have certain limitations; these methods typically involve continuous fluorescence excitation and imaging, which only provides two-dimensional information about imaged features (e.g., the location of disease). Conventional FGS does not provide important details, such as depth of a malignancy from the surface or the concentration of fluorescent contrast agent in tissue. Moreover, although techniques using near-infrared excitation provide deep tissue penetration,^13^ they do not provide practitioners with quantitative information on the three-dimensional (3D) location of target features.

Newer imaging sensors provide a critically important opportunity for advanced surgical instrumentation. In particular, large-format SPAD imagers have a wide range of applications ranging from surface topology mapping^14^^,^^15^ and automotive light detection and ranging (LiDAR)^16^ to time-resolved Raman spectroscopy, positron emission tomography, and super-resolution microscopy.^17^ In particular, SPAD imagers are highly valuable for real-time 3D imaging and fluorescence lifetime imaging at the macroscopic scale.^18^^,^^19^ This is due to their ability to precisely detect the timing of single photons with picosecond accuracy and virtually zero readout noise.^20^^–^^23^ In biomedical imaging, the presence of optically diffuse materials presents significant challenges for volumetric reconstruction.^24^ When optical photon transport is dominated by multiple strong scattering events, time-of-flight (TOF) histograms required for reconstruction are convolved with diffuse reflectance dynamics. This results in severe limitations to spatial accuracy. The distribution of optical photon mean free paths also encompasses absorption effects, which can pose difficulties for quantitative imaging of signal sources within optically diffuse media. However, unlike diffuse optical tomography,^25^^–^^28^ fluorescence depth sensing has the advantage of separating the interdependence of the optical properties of the medium from the fluorophore distribution, making it a more feasible approach to perform depth sensing.^29^

**Fig. 1:**
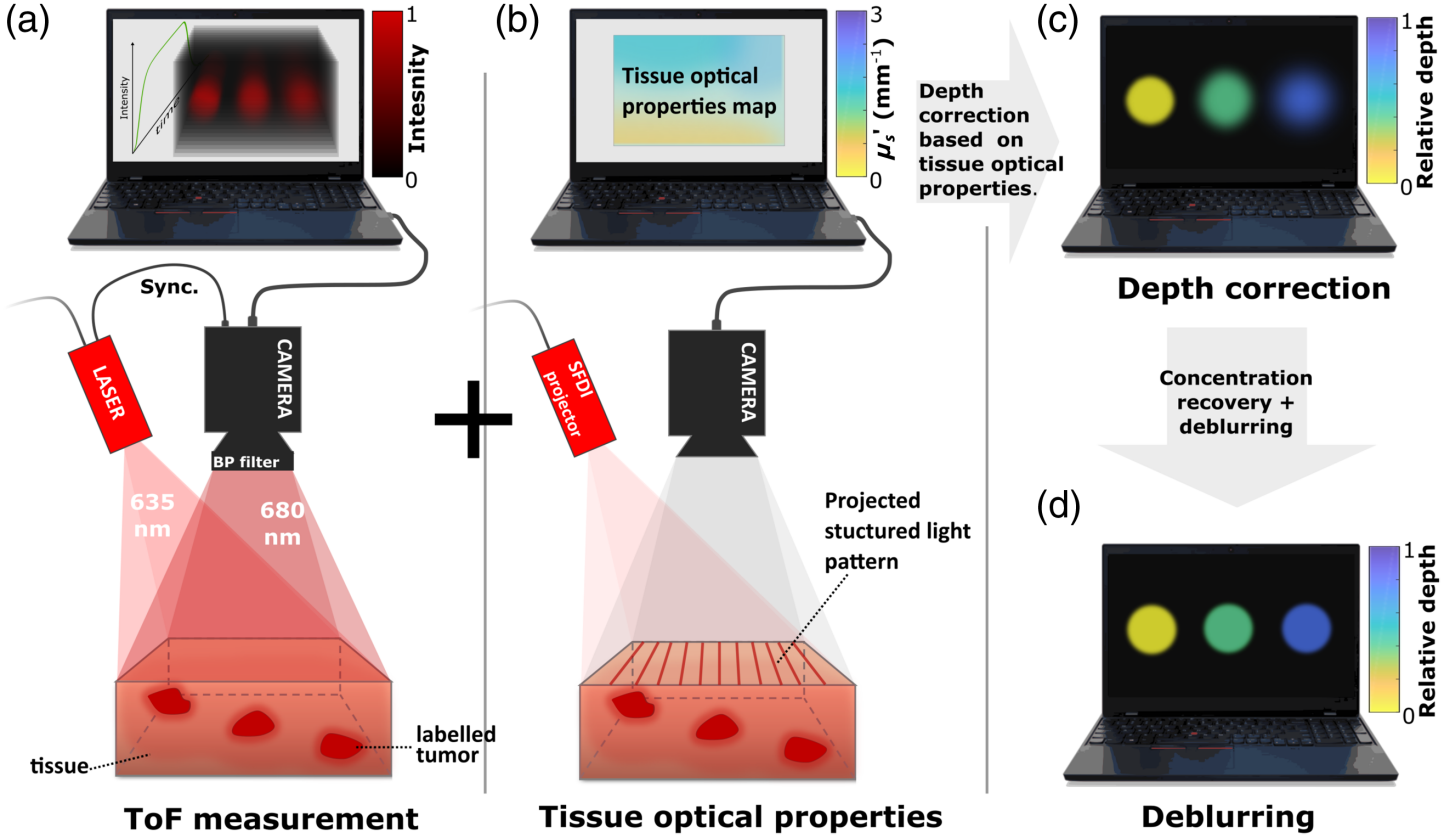
Study summary: (a) time-resolved acquisition of the fluorescent signal from the dyed inclusion embedded in a turbid medium at varied depths. (b) Measurement of the medium’s optical properties for calibration purposes. Combining these measurements enabled TOF correction and recovery of true depth. (c) Depth information and surface intensity enable the measurement of fluorophore concentration in each inclusion and (d) deblurring of the embedded inclusions. SFDI, spatial frequency domain imaging.

Recent developments in picosecond time-resolved, large-format SPAD imagers^30^ provide the ability to test this concept in a single-sensor, epi-illumination arrangement like that commonly used in medical imaging, microscopy, and metrology. Here a large-format SPAD sensor was coupled with a 20 MHz picosecond pulsed laser to acquire both fluorescence and reflectance images from turbid media samples containing fluorescent inclusions. The aim of this study was to develop a system capable of tumor depth sensing in turbid media. Figure 1 summarizes the different steps of the process. Diffuse reflectance is a reliable indicator of a medium’s optical characteristics, while the fluorescence signal conveys both the depth and optical properties of the fluorescent object. By measuring the TOF information along with diffuse reflectance, it is possible to reconstruct the location, shape, and concentration of the fluorophore in the object.

In this study, a range of tissue-like phantoms targeted with IRDye 680RD carboxylate (LI-COR Biosciences, Lincoln, Nebraska, United States) were imaged using the large-format SPAD camera. The fluorescence and reflectance time profiles (TPs) were analyzed by assuming that they were the outcome of the convolution of the instrument response function (IRF), probe fluorescent decay, and light source response of the observed diffuse light transport through the medium. Preliminary results from human biological tissue samples were also tested.

## 2 Materials and Methods

### 2.1 Setup

**Fig. 2:**
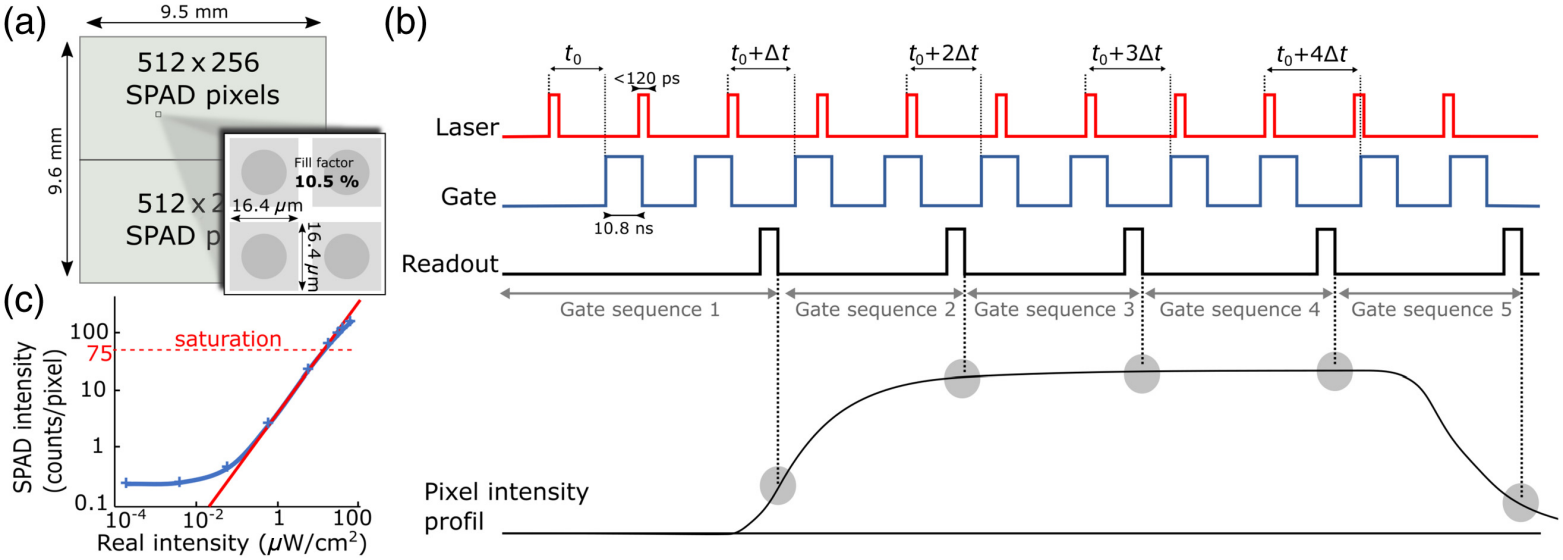
(a) Layout of the SwissSPAD2 sensor. (b) Time diagram of a laser pulse’s TP acquisition sequence. Both the laser and the camera are triggered by a 20 MHz clock signal. The number of gate sequences is defined by the user, and each of these sequences can be composed of a chosen number of frames (not shown here for clarity). (c) A representative curve depicting the SPAD sensor’s linear range for fixed acquisition parameters.

The imaging setup consisted of a 512×512  pixel SPAD array [Fig. 2(a)] (SwissSPAD2, EPFL, Neuchâtel, Switzerland) with 10.5% fill factor, coupled to a 635 nm 20 MHz pulsed laser (LDH-D-C-635M laser diode and PDL 828 driver, Picoquant, Germany) delivering ultrashort laser pulses (<120  ps). The main sync signal generated by the laser driver was converted to a +5 V TTL signal provided as an input to the camera’s field-programmable gate array (FPGA) (Opal Kelly XEM7360, Xilinx, San Jose, California, United States). The laser was operated at maximum intensity, providing an optical power density of $160\;\mu W/{\mathrm{cm}}^{2}$ in the target plane. The SPAD device and its FPGA were mounted in a custom aluminum case with forced-air cooling. A C-Mount was attached to the case and aligned with the SPAD sensor to relieve stress on the sensor’s printed circuit board. A 50 mm, 1:1.8 D lens (Nikkor, Nikon, Tokyo, Japan) was used in a first experiment and a 50 mm F0.95 lens (TV-lens, Lenzar, Jupiter, Florida, United States) in a second experiment to increase light collection capabilities of the system.

For each experiment, the SPAD camera was placed directly above the target plane at a working distance of 50 cm. The laser beam was provided as input to the imaging setup using a fiber with a lens and a top hat diffuser fixed on the fiber terminus. The laser was placed in a way to reduce its angle with the normal to the sample’s surface while ensuring that it did not obstruct the camera’s field of view. Data transfer was achieved via USB from the FPGA to a dedicated imaging system computer.

#### 2.1.1 Camera acquisition specifications

The SPAD camera operated in a time-gated mode to image temporal profiles of the laser pulse backscattered from the medium. An acquisition gate width of 10.8 ns was used for this study, and the time-gate step (i.e., the delay between subsequent gate positions) was set to 17.857 ps. For each experiment, a different gate offset (i.e., delay) from the sync pulse [parameter $t_{0}$ in Fig. 2(b)] was selected. To make use of the full dynamic range of the sensor and avoid saturation, the exposure time per binary frame was set at 4  μs. Each gate sequence consisted of 256 binary frames, resulting in 8 bit gray-scale images. Figure 2(b) shows a time diagram of a laser pulse time response acquisition sequence. The red signal shows the laser pulse corresponding to the 20 MHz sync signal. The rising edge of this signal triggers the camera gate (blue signal) after a delay of $t_{0}+\Delta t$, where Δt is the time-gate step between two subsequent gate positions, fixed to 17.857 ps. After each gate sequence, Δt is incremented. A gate sequence is composed of 256 binary frames [not shown in Fig. 2(b)], and each time scan consists of a total of 1400 gate sequences, corresponding to an overall 25 ns time window. The frame readout is performed in 10  μs in a rolling shutter mode. Due to a limited number of input/output pins on the FPGA, only 256 rows and 472 columns of the SPAD array were available in this study. Note that utilizing two separate FPGA boards enables the full use of the 512×512  pixel array.

To characterize the sensitivity range of the SPAD sensor, we measured the photon counts/unit time/pixel, which is characterized as a function of the excitation source intensity in $\mu W/{\mathrm{cm}}^{2}$. Results are displayed in Fig. 2(c). This was done using a constant illumination power density and a set of neutral density (ND) filters of known optical density placed in front of the laser source to vary the laser output power in a controlled fashion. During each measurement, care was taken to remain within the linear range of the sensor’s dynamic range as defined by the sensitivity curve shown in Fig. 2(c).

#### 2.1.2 Data acquisition

The time-resolved acquisition sequence described above results in a 3D image stack with dimensions of 256×472×1400, corresponding to the spatial rows, spatial columns, and time points, respectively. These data were then analyzed by selecting spatially averaged regions of interest (ROIs) with constant sizes, across the entire time scan, resulting in 1×1400 TPs. The following section details how these TPs were acquired and processed.

##### Time profile generation

**Fig. 3:**
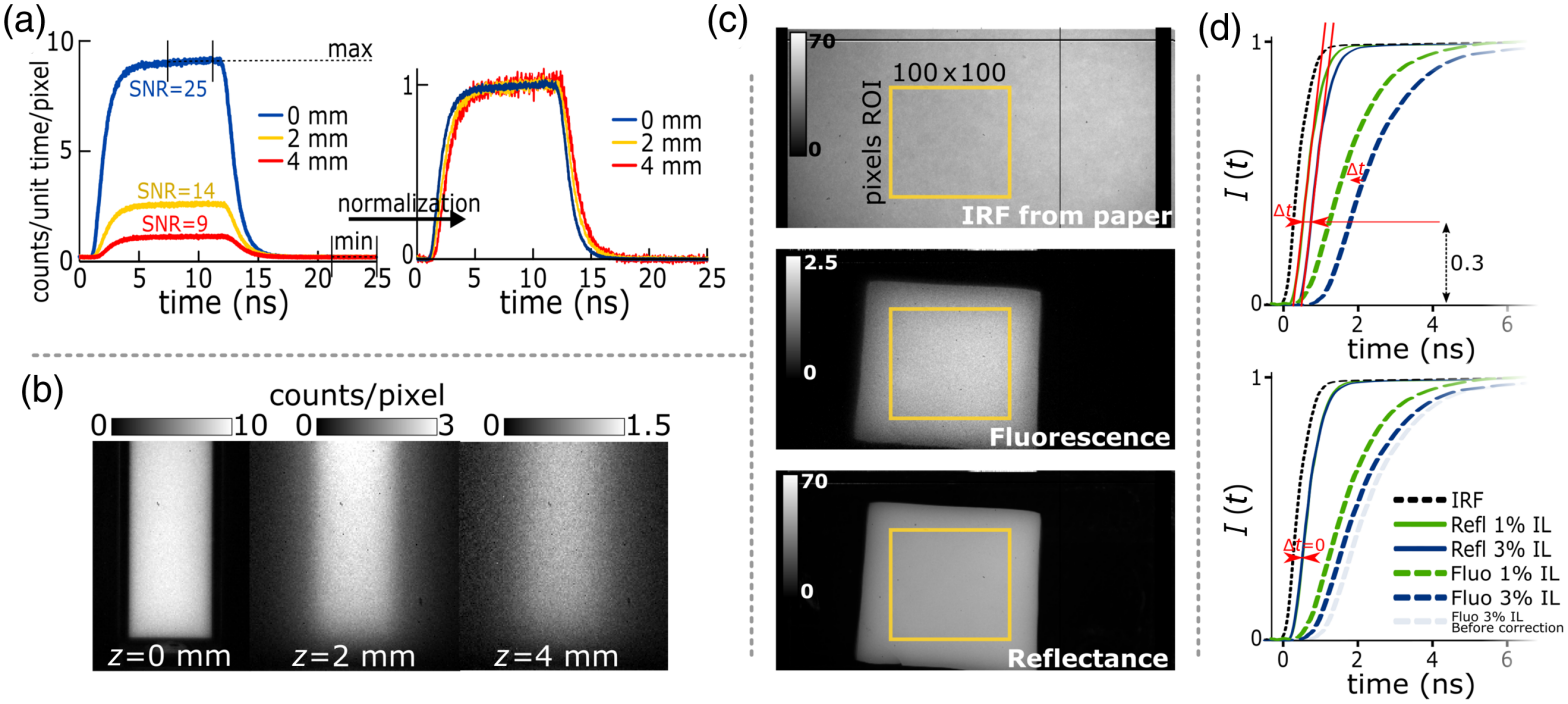
(a) Fluorescence TPs from inclusions in 3% Intralipid solution at depths of 0, 2, and 4 mm, normalized to maximum intensity for comparison. For each plot, the SNR is displayed for an ROI corresponding to an 8×8 binned pixel area. (b) Corresponding intensity images showing the spatial blur and signal loss with increasing inclusion depth. (c) From top to bottom, IRF from white paper, fluorescence intensity image, and reflectance intensity image averaged over 100 frames during the pulse; the co-registered ROIs enable fluorescence correction. (d) How fluorescence TPs are corrected by corresponding reflectance TPs. Setup changes induce time shifts in the TPs, and correcting for this allows accurate comparison of TPs between acquisitions. IRF, instrument response function.

Depending on the signal-to-noise ratio (SNR) and lateral spatial resolution desired, TPs were generated over varying ROI sizes. Increasing the TP ROI size increases SNR at the cost of lower spatial resolution and vice versa. For each pixel, a TP corresponded to the number of photons detected as a function of time. Each TP was normalized to its maximum intensity, corresponding to the average value of the 200 most intense frames, and to its minimum intensity, corresponding to the average value of the 200 last frames where no fluorescent signal was detected; this normalization mapped TP values between the average maximum and minimum to the range of [0, 1] [see Fig. 3(a)].

##### Instrument response function

The IRF of the SPAD camera provided a reference for data fitting and correction in each experiment. The IRF served to rectify pixel temporal inconsistencies as well as variations in illumination across the field of view. Each fluorescence or reflectance data TP fitting was derived using a corresponding ROI on the IRF time scan [see Fig. 3(c)]. In each setup configuration, the IRF was acquired to account for the setup geometry, because any adjustment in setup geometry modified the IRF both spatially, because of the laser angle, and in time, because of the camera-to-sample working distance. The IRF was acquired using a sheet of white paper placed in the imaging plane of the setup. Here this paper sheet was considered a Lambertian surface, allowing for temporal correction of the fluorescence and reflectance signals over the entire field of view.

##### Fluorescence data

IRDye 680RD was excited using a 635 nm wavelength laser, corresponding to the dye’s first absorption Q-band. A 697 nm band-pass filter with 75 nm bandwidth and OD>6 (Edmund Optics, Barrington, New Jersey, United States) separated the emission signal from the excitation wavelength.

##### Reflectance data

Reflectance data were acquired in the same way as fluorescence data, except that the 697 nm band-pass filter was removed, and linear polarizing filters were placed on the laser fiber optic output and on the front of the camera lens. The polarizers were offset by 90 deg. It is well-known that polarized light remains polarized after undergoing specular reflection, whereas polarized light loses its polarization when passing through turbid media. Therefore, the use of crossed polarizers minimized specular reflections from the surface of each sample. This was especially important when working with surfaces that exhibited high levels of specular reflection, such as solutions and gelatin phantoms, as these reflections can otherwise overpower the useful signal originating from backscattered light. Additionally, the polarizers functioned as ND filters to account for the absence of the spectral filter and to prevent sensor saturation.

##### Acquisition sequence

For each imaging session, an IRF was acquired. All data collected during the session were corrected using the same IRF. To compensate for samples’ surface-to-sensor distance changes and surface geometry, each fluorescence TP acquisition was coupled with a reflectance TP acquisition using the same settings. To facilitate data collection for untrained operators, a graphical user interface (GUI) was implemented using MATLAB (vR2018a, MathWorks Inc., Natick, Massachusetts, United States). The GUI allowed for automatic acquisition of a set of five fluorescence TPs and five reflectance TPs, with a break in between allowing the user to switch the filters. Raw data were automatically saved for subsequent analysis. An entire acquisition sequence using the GUI took <10  s.

### 2.2 Sample Preparation

#### 2.2.1 Intralipid and fluorescence phantoms

The first experiment used a $1\times 1\times 4\;{\mathrm{cm}}^{3}$ sample containing a 10  μM mixture of IRDye 680RD Carboxylate (Li-COR bioscience, Lincoln, Nebraska, United States) in a turbid solution made of IL (Intralipid 20%, Fresenius Kabi, Bad Homburg, Germany) dissolved in phosphate buffered saline (PBS) (DPBS 1×, Corning Inc., Corning, New York, United States). Samples were prepared at constant dye concentration with IL concentrations of 0.5%, 1.0%, 1.5%, 2.0%, and 3.0%. The fluorescent samples were immersed in undyed matching solutions of IL. IL matching solution was added gradually above the samples, and images were acquired with respect to increasing inclusion depth. Fluorescence and reflectance TPs were acquired at inclusion depths of 0 to 5 mm in 1 mm steps. Figure 5(c) illustrates this setup.

#### 2.2.2 Tissue-like phantoms

In the second experiment, solid tissue-like phantoms were prepared using a mixture of 10% w/v gelatin from porcine skin (Sigma-Aldrich, St. Louis, Missouri, United States) in PBS, 1% bovine whole blood in ACD (Lampire Biological Laboratories, Pipersville, Pennsylvania, United States), and varied IL concentrations. The stock mixture at each IL concentration was divided into two parts, and fluorescent dye was added to one part.

##### Variable tissue-like medium thickness using 1 mm slabs

Solid tissue-like phantoms were prepared using a mixture of porcine gelatin, 1% bovine blood, and IL at concentrations of 1%, 2%, and 3%. Each of these three solutions was split into two respective containers, and IRDye 680LT (LI-COR Biosciences, Lincoln, Nebraska, United States) was added at a 50 nM concentration in half of the preparations before solidification. Six solid phantoms were obtained: two at each IL concentration, one containing fluorescent dye and the other without dye. These phantoms were then cut into 1 mm thick (±0.05  mm) slabs using a vibratome (Leica VT1200S, Leica Biosystems, Wetzlar, Germany), resulting in ten $1\times 25\times 25\;{\mathrm{mm}}^{3}$ phantoms from each of the six blocks. To create a phantom, a set of slabs with matching Intralipid concentrations were stacked on top of each other in a specific order: five dyed slabs were followed by five undyed slabs, arranged from bottom to top, as shown in Fig. 7(a). This allowed for the imaging of dyed slabs covered by nonfluorescent, tissue-like material at a range of controlled thicknesses by removing the undyed slabs one by one from the top of the stack.

##### Tissue-like medium with cylindrical inclusions

To mimic a fluorescent inclusion within tissue, 4 cm long plastic tubes, each 1 cm in diameter, were filled with gelatin/blood/IL mixture and dyed with IRDye 680RD carboxylate at 10  μM concentration. After the dyed mixture solidified, the tubes were sealed and submerged at different depths in undyed, unsolidified mixture with matching optical properties. To accomplish this, a first layer of undyed mixture was added into a mold. Once solidified, a tube was added on top of the first layer and thereafter covered with more undyed mixture. The same step was then repeated with a second tube to finally end with a 16 mm thick phantom with fluorescent inclusions at depths of 2.5 and 5.6 mm [Fig. 9(d)]. Once imaged, the phantom was sliced in half, and the cross section was imaged with a closed-field, commercial fluorescence imaging system (Odyssey CLx, Li-COR Biosciences, Lincoln, Nebraska, United States) using its 700 nm acquisition channel. By this method, the surface-to-inclusion depths were precisely measured [Fig. 9(d)].

#### 2.2.3 Imaging of fluorescently labeled human tumor resections

To test our system in a clinical setting, two representative slices from two freshly resected head and neck tumors were imaged using the same settings as for the previous experiments (Sec. 2.2.1). The patients involved were enrolled in a clinical study to examine the value of ABY-029 (an antiepithelial growth factor receptor Affibody molecule labeled with IRDye 800CW)^31^ for demarking tumor tissue (ClinicalTrials.gov Identifier: NCT03282461). All procedures followed an approved Institutional Review Board protocol at Dartmouth Hitchcock Medical Center (DHMC), Lebanon, New Hampshire, United States. Each patient in the study was administered 180 nanomoles of solubilized ABY-029 via intravenous injection 2 to 4 h prior to surgery. Surgery proceeded according to standard-of-care; no fluorescence imaging was performed intraoperatively, and fluorescence imaging results did not in any way impact clinical decision making. Resected tissues were breadloafed into sections in the gross pathology laboratory at DHMC according to standard-of-care. The final step for the ABY-029 clinical study involved fluorescence imaging of representative tissue sections using a closed-field, commercial fluorescence scanning imager (Odyssey CLx, Li-COR Biosciences, Lincoln, Nebraska, United States) for complete measurement of signal on the exposed surfaces. The large-format SPAD camera imaging for the present study was conducted in the gross pathology laboratory, prior to imaging in the closed-field scanner.

### 2.3 Data Processing

#### 2.3.1 Time profile fitting

**Fig. 4:**
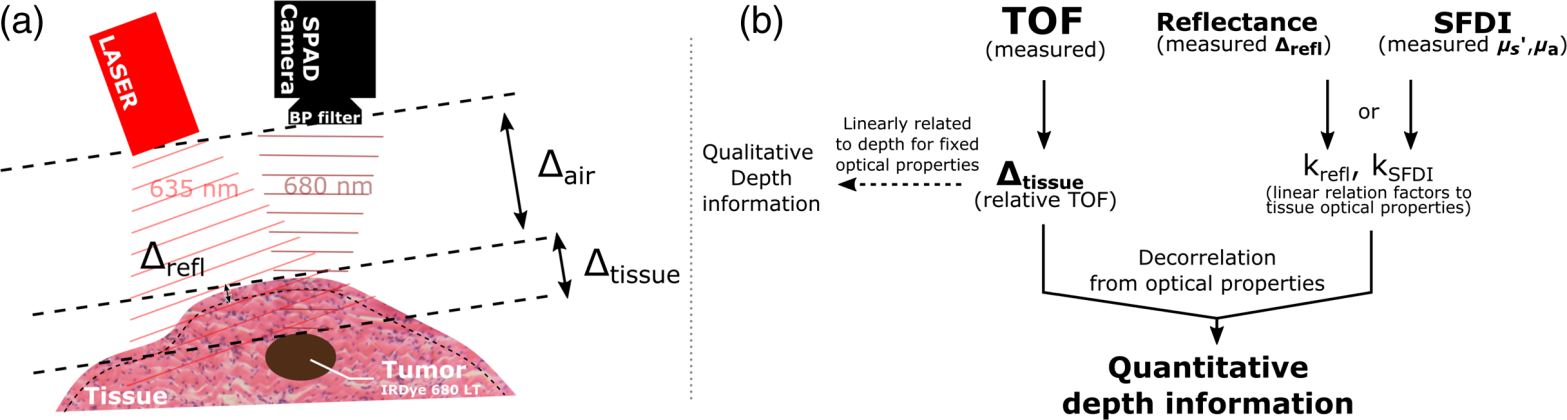
(a) Schematics of the setup with wavefronts represented by dotted lines to delimitate TOF paths. (b) Flowchart of depth calibration using TOF measurements and either reflectance measurements or SFDI measurements to correct for tissue optical properties.

To obtain accurate TOF measurements, it was necessary to fit both fluorescence and reflectance TPs to an analytical model. The analytical model was derived by examining the various stages of how light pulses interact with the medium and different layers of the geometry. The IRF was characterized as the TP resulting from a laser pulse propagating through a negligible thickness of nonscattering medium (i.e., air), reflecting off a Lambertian surface, and subsequently propagating back to the detector. In the case of a laser pulse propagating through a turbid medium, the TP is altered, and a term representing the medium transfer function must be incorporated. This is expressed by the following equation:

$$\mathrm{TP}=\mathrm{IRF}*H_{\text{medium}}{*\Delta }_{\text{air}},$$ (1)

where TP is the resulting time profile registered by the sensor, IRF is the instrument response function described above, $H_{\text{medium}}$ is the medium transfer function, $\Delta_{\text{air}}$ is the delay corresponding to light’s travel through air [see Fig. 4(a)], from the laser head to the tissues and back (negligible), and * is the convolution operator.

If the pulse train, in addition to traveling through a turbid medium, encounters a Stokes shift due to a fluorescent emitter, another convolution must account for the phenomenon:

$$\mathrm{TP}={\mathrm{IRF}*H}_{\text{medium}}*e^{-t/\tau_{\mathrm{fluo}}}{*\Delta }_{\text{air}},$$ (2)

where τ is the fluorescent lifetime of the probe assuming a monoexponential response.

##### Reflectance data

Reflectance data can be fitted using Eq. (1), because reflectance results primarily from light interactions with the superficial layer of a turbid medium,^32^ and it can be assumed that the fluorescent contribution is negligible in terms of intensity. In this study, we endeavored to identify a mathematically simple yet effective expression that could enhance computational efficiency. The medium transfer function $H_{\text{medium}}$ can be effectively modeled through a Gaussian function, as empirically substantiated by the reflectance fitting results. The mean goodness of fit for reflectance data was found to be 0.997±0.011, providing strong evidence for the appropriateness of the Gaussian function in capturing the characteristics of $H_{\text{medium}}$. The TP fitting model can therefore be described as follows:

$$I_{\mathrm{fit}}(t)=\mathrm{IRF}*\frac{A}{\sigma \sqrt{2\pi }}e^{\frac{-{\left(t-\Delta_{\text{tissue}}\right)}^{2}}{2\sigma^{2}}},$$ (3)

where $I_{\text{fit}}$ is the analytical model fitting the reflectance TP, dependent on time t, and A, $\Delta_{\text{tissue}}$, and σ are the amplitude, mean, and standard deviation, respectively, of the Gaussian distribution modeling the medium transfer function. In practice, $\Delta_{\text{air}}$ is corrected by the IRF. The three parameters A, $\Delta_{\text{tissue}}$, and σ were set as variables, and $I_{\text{fit}}$ was fitted to the raw data using a least squares method.

##### Fluorescence data

**Fig. 5:**
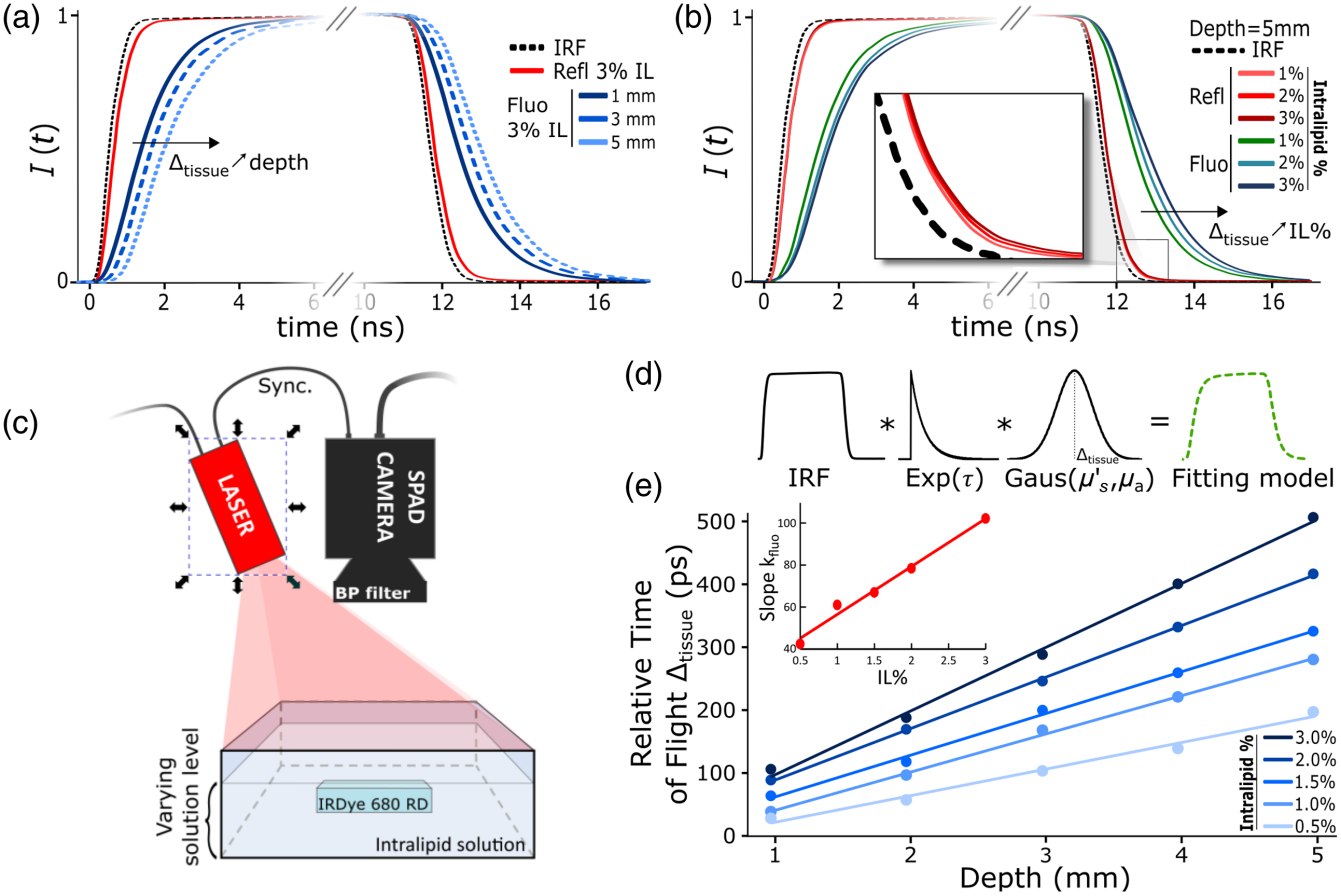
(a) Influence of depth on the fluorescence TP’s shift and dispersion. The figure shows the fluorescent profiles at three different depths, as well as the reflectance TP for a $1\times 1\times 4\;{\mathrm{cm}}^{3}$ IRDye 680RD carboxylate inclusion embedded in a 3% IL aqueous solution. (b) Influence of the medium’s scattering coefficient on the fluorescence TP shift and dispersion. The influence on the reflectance TP is less pronounced but can be seen in the inset window. (c) Schematic of the setup showing the fluorescent cuvette immersed in an Intralipid solution. (d) The TPs analytical fitting model is the result of the convolution between the IRF, an exponential function corresponding to fluorescence decay, and a Gaussian function that reflects the medium’s transfer function. (e) Relative TOF $\Delta_{\text{tissue}}$ compared to inclusion depth at varying IL concentration. $\Delta_{\text{tissue}}$ corresponds to the Gaussian’s central value displayed in (d). The red plot represents the linear relation between the slope of each of the five TOF curves as a function of IL concentration. IL, Intralipid.

Following similar logic, the backscattered fluorescence TP fitting can be modeled using Eqs. (2) and (3), where the fluorescent term can be described as follows:

$$I_{\mathrm{fit}}(t)=\mathrm{IRF}*\frac{A}{\sigma \sqrt{2\pi }}e^{\frac{-(t-\Delta_{\mathrm{tissue}}{)}^{2}}{2\sigma^{2}}}*e^{-t/\tau },$$ (4)

where τ is the fluorescence lifetime of the probe. For all the fitting done in this paper, τ was set to 0.7 ns, as measured using time correlated single-photon counting lifetime measurements (Fluoromax, Kyoto, Japan). This simple model is illustrated in Fig. 5(d).

#### 2.3.2 Time profiles comparison

**Fig. 6:**
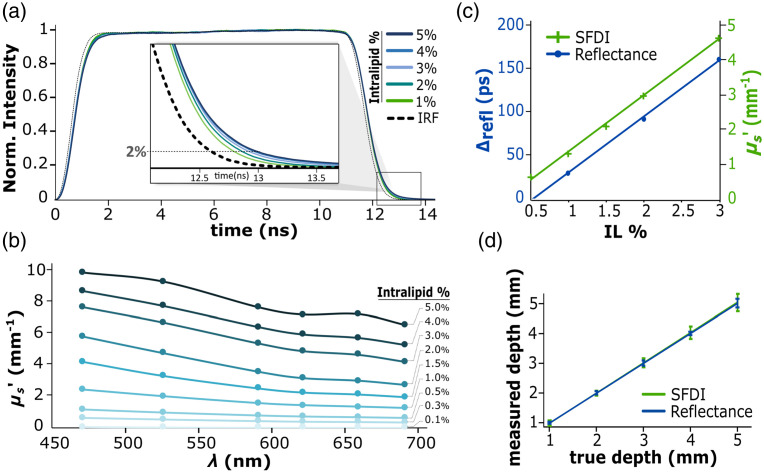
(a) Reflectance TPs with respect to IL concentration. Although temporal variations are small relative to the overall pulse length, the inset window highlights how IL concentration impacts the falling edge of each pulse. (b) SFDI-derived reduced scattering coefficient spectra for different IL concentrations. (c) Illustration of the linear relation between measurements from (a) and (b) and IL concentration. $\Delta_{\mathrm{refl}}$ represents the time from reflectance TPs at 2% of the normalized pulse. (d) Agreement is observed between SFDI-based and reflectance-based depth measurement calibration.

To compare results from different acquisitions—i.e., experiments where modifications were made to the setup or where samples were replaced (as was the case for the liquid phantom and the gelatin slabs experiments), it was necessary to ensure that any changes did not introduce bias. Thus to accurately compare fluorescent TPs from different acquisitions, each fluorescent TP was time-corrected to its corresponding reflectance TP’s rising edge. Raw reflectance TP rising edges were fitted with linear functions, as shown in Fig. 3(d), which were then used as a time reference. Figure 3(d) provides an illustration of the fluorescence TP before and after correction. The red lines represent the linear fit of the reflectance rising edge, and Δt represents the time delay applied to correct the fluorescence TPs. The corresponding IRF is shown for reference. To enhance clarity, all TP data shown in Figs. 3, 5, and 6 were smoothed with a 35 element (i.e., 623 ps) moving average filter.

#### 2.3.3 TOF map generation

The primary objective of employing a large-format SPAD array is to spatially resolve TOF measurements over a large array of pixels. Accordingly, the subsequent section outlines the process by which TOF maps were generated through analysis of data obtained from a single-phantom acquisition. This methodology was implemented for both the gelatin phantom tubes and clinical imaging experiments in this paper.

##### Gelatin phantom: plastic tube inclusion

Time scans of the phantoms were acquired, and 8×8  pixel binning was implemented to reduce processing time and reduce the effect of thermal and shot noise, resulting in a 32×59  pixel image. Each pixel’s TP was normalized to its maximum intensity and average background level, as described in Sec. 2.1.2 and Fig. 3(a). Each pixel’s TP was fitted according to the method described in Sec. 2.3.1. Both $\Delta_{\text{tissue}}$ and σ fitting parameters were then plotted spatially [Figs. 9(a) and 9(b)]. The TOF maps represent the spatial distribution of the fitting parameter $\Delta_{\text{tisue}}$ for a given pixel size. To mitigate the effects of noise resulting from the lack of signal, the TOF maps were subjected to a correction process based on their corresponding fluorescence intensity image.

##### Tumor resection

The method for obtaining the TOF map for the head and neck resection specimens was consistent with that described in the preceding section. Due to the low SNR of this dataset (SNR∼1.4), an 8×8 binned pixel image was utilized to generate the TOF map. Here SNR is defined as the ratio between the mean signal and the standard deviation of a background area adjacent to the fluorescent object, where no light emission occurs. All images presented in this study underwent darkfield subtraction, utilizing an image acquired under identical camera settings in total darkness. Additionally, for the tumor resection, five time-scan measurements were performed and averaged to further reduce the effect of thermal and shot noise. This resulted in an increased SNR of ∼3. Furthermore, a fluorescence intensity map was produced by averaging 300 frames to enhance contrast and reduce noise levels. Both TOF map and fluorescence distribution images were superimposed onto the reflectance intensity image of the tumors to provide a more comprehensive depiction of the fluorescence distribution in the tissue.

### 2.4 Depth Calibration/Correction

It is important to note that an absolute TOF measurement may not necessarily reflect an accurate distance traveled by photons. This is because the TOF of backscattered photons is influenced by the optical properties of the turbid medium. To obtain precise depth measurements, it is essential to calibrate the absolute TOF measurements in accordance with the medium’s optical scattering and absorption properties. The two methods described below were evaluated for the purpose of performing this calibration and are described in Fig. 4(b).

#### 2.4.1 Medium optical properties

##### Time-resolved reflectance

The first method involved time-resolved imaging of reflectance, which provides information about the optical properties of the medium provided that a sufficiently high time resolution is used. In this experiment, optical properties of the medium were quantified through the analysis of reflectance TPs variations. To this end, reflectance TPs were acquired under the same conditions as previously described. TPs were recorded from 1%, 2%, 3%, 4%, and 5% IL solutions, as well as from porcine gelatin phantoms containing 1% blood and 1%, 2%, and 3% IL. These phantoms are discussed in more detail in Sec. 3. To enable comparison between the different samples, the reflectance TPs were aligned based on their falling edge. The shifts in the various curves were subsequently compared using a 2% threshold of the maximum intensity, as illustrated in Fig. 6(a).

##### Spatial frequency domain imaging

The second method employed quantitative structured light imaging to determine the optical properties of the medium. Specifically, spatial frequency domain imaging (SFDI) was performed using a commercially available and validated system (Reflect RS, Modulim, Irvine, California, United States). Optical properties were quantified at each of eight wavelengths (471, 526, 591, 621, 659, 691, 731, and 851 nm) using five spatial projection frequencies (0.00, 0.05, 0.10, 0.15, and $0.20\;{\mathrm{mm}}^{-1}$). These measurements allowed for the recovery of pixel-level absorption ($\mu_{a}$) and reduced scattering ($\mu_{s}^{\prime }$) coefficients from the target medium at each wavelength.

#### 2.4.2 Depth calibration

Depth calibration was achieved using data obtained from the two methods described above. These methods enabled the derivation of a linear relationship between IL concentration and either relative time or reduced scattering coefficient [see Fig. 6(c)], respectively:

$$\Delta_{\mathrm{refl}}=k_{\mathrm{refl}}\times \mathrm{IL}+c_{\mathrm{refl}},$$ (5)

and

$$\mu_{s}^{\prime }=k_{\mathrm{SFDI}}\times \mathrm{IL}+c_{\mathrm{SFDI}},$$ (6)

where $\Delta_{\mathrm{refl}}$ is the relative time shift of normalized reflectance TPs at 2% [as described in the previous section and Fig. 4(a)], and $\mu_{s}^{\prime }$ is the reduced scattering coefficient obtained using SFDI. Both these parameters are related to the IL concentration by a linear relation, where $k_{\mathrm{refl}}$ and $k_{\mathrm{SFDI}}$ are slopes, and $c_{\mathrm{refl}}$ and $c_{\mathrm{SFDI}}$ are constants.

Additionally, TOF from fluorescence backscatter can also be related to inclusion depth by a linear expression [see Fig. 5(e)] as follows:

$$\Delta_{\text{tissue}}=k_{\mathrm{fluo}}\times \text{Depth}+c_{\mathrm{fluo}},$$ (7)

where $\Delta_{\text{tissue}}$ corresponds to the relative TOF obtained from the Gaussian fitting parameter (see Sec. 2.3.1), $k_{\mathrm{fluo}}$ is the slope of the linear fit, Depth is the depth of the inclusion, and $c_{\mathrm{fluo}}$ is a constant.

Here the linear relationship between TOF and depth is dependent on the level of scattering in the medium. Therefore, a relationship can be established between $k_{\mathrm{fluo}}$ and the IL concentration, as shown in the following equation:

$$k_{\mathrm{fluo}}=k_{\mathrm{corr}}\times \mathrm{IL}+c_{\mathrm{corr}},$$ (8)

where $k_{\mathrm{fluo}}$ is the fitting parameter from Eq. (7), $k_{\mathrm{corr}}$ is the slope of the linear fit later used for data calibration, and $c_{\mathrm{corr}}$ is a constant. Combining Eqs. (7) and (8) with either Eq. (5) or Eq. (6), depending on the calibration method, the depth of the inclusion can be, respectively, expressed as

$$\text{Depth}=(\Delta_{\text{tissue}}-c_{\mathrm{fluo}})/(k_{\mathrm{corr}}\times \frac{\Delta_{\mathrm{refl}}-c_{\mathrm{refl}}}{k_{\mathrm{refl}}}+c_{\mathrm{corr}}),$$ (9)

or

$$\text{Depth}=(\Delta_{\text{tissue}}-c_{\mathrm{fluo}})/(k_{\mathrm{corr}}\times \frac{\mu_{s}^{\prime }-c_{\mathrm{SFDI}}}{k_{\mathrm{SFDI}}}+c_{\mathrm{corr}}).$$ (10)

#### 2.4.3 Intensity correction

Once quantitative depth information is known, it becomes possible to correct the measured intensity of light at the surface of a turbid medium to recover the original volumetric luminance distribution throughout the medium. This is done using a simple equation^33^ accounting for losses caused by photon absorption and diffusion as light travels a distance z through the medium:

$$I=L\times \exp(-\mu_{\mathrm{eff}}2z),$$ (11)

where I is the measured surface intensity, L is the original luminance at depth z, and $\mu_{\mathrm{eff}}$ is the effective scattering coefficient^34^ expressed as

$$\mu_{\mathrm{eff}}=\sqrt{3\mu_{a}(\mu_{a}+\mu_{s}^{\prime })}.$$ (12)

## 3 Results

### 3.1 Liquid Phantom

The depth sensing system was first tested on a simplified tissue phantom model. The phantom consisted of a cuvette immersed in turbid solutions with varied scattering coefficients as shown in Fig. 5(c). TPs shown in Figs. 5(a) and 5(b) were obtained as described in Sec. 2.3.1 using a 40×40  pixel ROI. Figure 5(a) shows the influence of the inclusion depth on the fluorescence TPs recorded by the SPAD camera. These pulses are plotted next to corresponding IRF and reflectance TPs. Because the reflectance TPs were very similar for the various inclusion depths, only one reflectance curve is displayed in Fig. 5(a) for clarity. The plot shows that the fluorescence TP is affected in two ways as the inclusion depth increases: first, the TPs shift toward the right as depth increases, i.e., $\Delta_{\text{tissue}}$ increases, and second, the TP becomes increasingly dispersed with depth, i.e., σ increases. The pulse spread is not clearly visible in these figures but will be described in more detail in Sec. 3.5. Figure 5(b) shows the influence of the medium’s optical scattering level at 5 mm depth, expressed here in terms of IL concentration, IL%, on the pulse. The impact of IL% on the pulse is similar to that observed in Fig. 5(a): an increase in scattering coefficient leads to greater temporal shifts and dispersion of the pulse. The coefficient $\Delta_{\text{tissue}}$ expressed in Figs. 5(a) and 4(b) corresponds to the Gaussian’s mean value fitting coefficient. To aid the comprehension of fluorescent pulse fitting, an illustration of the convolution product is presented in Fig. 5(d). The model used to fit raw data results from the convolution of the IRF, an exponential decay representing the fluorescent re-emission, and a Gaussian function describing the medium’s effect on the light pulse TP. Later in this paper, we will demonstrate that $\Delta_{\text{tissue}}$ is the most accurate reporter of TOF (see Sec. 3.5). Figure 5(e) finally summarizes the results obtained from five different datasets, each corresponding to an IL% ranging from 0.5% to 3.0%. TOF is expressed as $\Delta_{\text{tissue}}$ and is compared to the inclusion depth. A linear relationship can be observed between TOF and depth for a given IL%. Once fitted, the slopes $k_{\mathrm{fluo}}$ of these five curves can be plotted against IL%, revealing a linear trend.

### 3.2 System Calibration

This section focuses on absolute TOF measurement calibration by considering the optical properties of the bulk medium. Figure 6 illustrates the results of TOF calibration using two different methods for assessing the optical properties, as described above. The first method [Fig. 6(a)] utilizes reflectance TP measurements. Although reflectance TPs are less affected by tissue optical properties than fluorescence TPs, some variation can still be observed for varying IL concentrations. Figure 6(a) shows reflectance TPs obtained from IL in aqueous solution with 1%–5% IL concentrations, along with the corresponding IRF. All five reflectance TPs were aligned on their rising edge, as described in Sec. 2.3.2, and compared at a 2% threshold of the normalized pulse, as described in Sec. 2.4.1. The corresponding time value is denoted as $\Delta_{\mathrm{refl}}$, which exhibits a linear relationship when plotted as a function of IL%. A closer look is provided in the inset window in Fig. 6(a). The portion of this curve used for calibration is shown in Fig. 6(c) as the blue line. It should be noted that $\Delta_{\mathrm{refl}}$ is a relative time value, where $\Delta_{\mathrm{refl}}=0$ was chosen arbitrarily at IL%=0.5.

The second method used for characterizing the medium’s optical properties was SFDI. Figure 6(b) shows the reduced scattering coefficient $\mu_{s}^{\prime }$ corresponding to various IL solutions in aqueous solution, measured with the SFDI imaging system. $\mu_{s}^{\prime }$ at 659 nm is plotted as a function of IL% in Fig. 6(c), showing only the range of IL% used in the liquid phantom experiment (Sec. 2). The linear relationship between $\mu_{s}^{\prime }$ and IL% is evident.

Finally, Fig. 6(d) shows the data obtained from the IL solution phantom presented in Fig. 6(e), corrected using the reflectance data (blue curve) and SFDI-based measurements (green curve), respectively. Both lines represent the trendline of the average corrected data, and the error bars represent the standard deviation of the five measurements. These data are corrected using Eqs. (10) and (11), respectively.

### 3.3 Gelatin Slabs Phantom

**Fig. 7:**
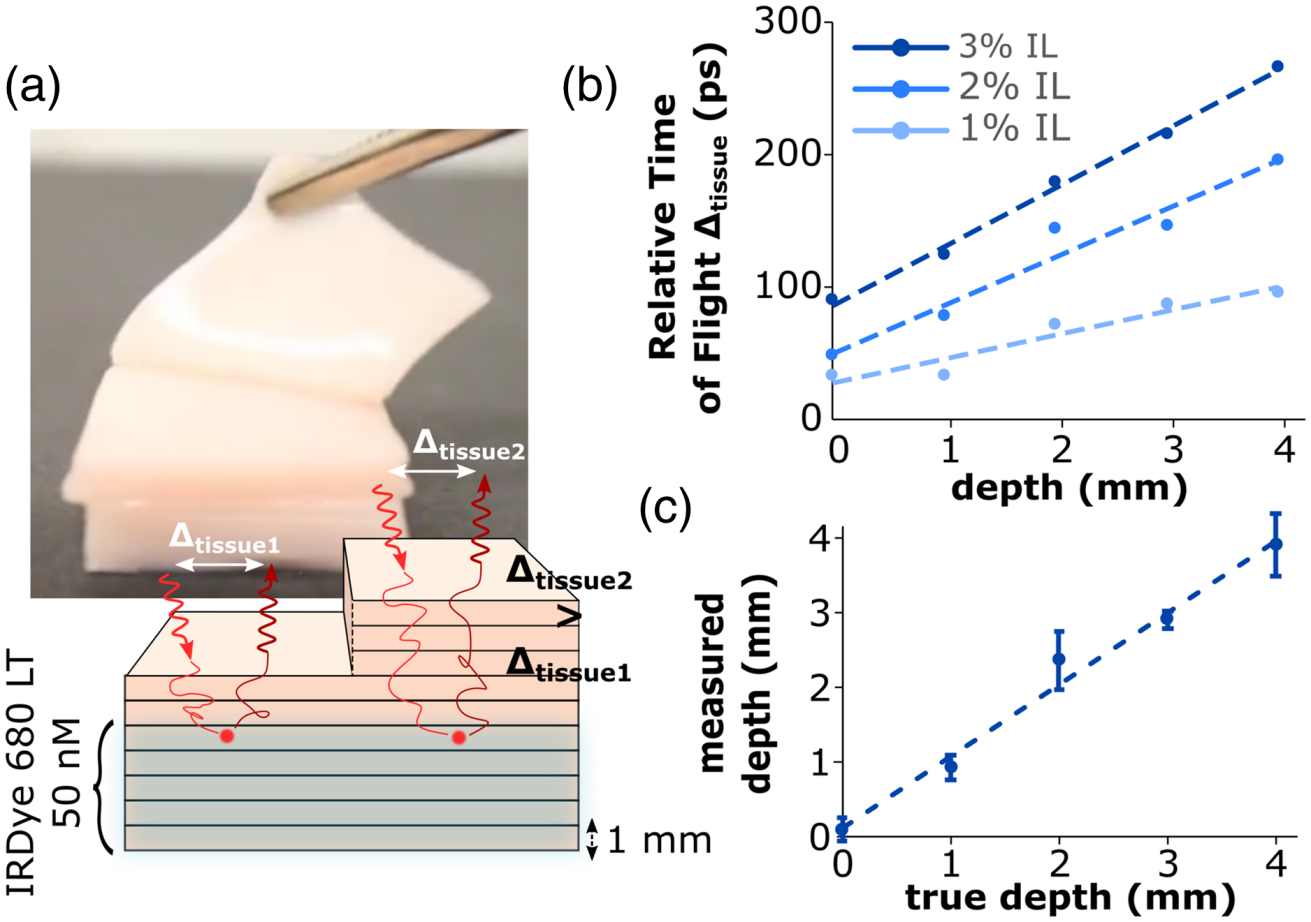
(a) Illustration of the gelatin slabs phantom, including a drawing that shows the layering of the dyed and undyed slabs as well as the time difference between photons traveling through different thicknesses of the medium. The photograph illustrates the process of peeling away slabs to adjust the inclusion depth. (b) Relative TOF $\Delta_{\mathrm{tissue}}$ versus inclusion depth at varying IL concentrations from the gelatin slabs phantom. (c) Depth correction obtained using SFDI data from the varying IL phantoms.

To create a more controlled and realistic scenario for imaging fluorescent inclusions at varied depths, we conducted a third experiment using gelatin slabs that were 1 mm thick and had tissue-like optical properties. These slabs were stacked on top of each other, as illustrated in Fig. 7(a). The phantom setup allowed for precise control of the inclusion depth by adding or removing layers one by one. The schematic from Fig. 7(a) shows two scenarios, one representing a 2 mm thick layer above the inclusion ($\Delta_{\text{tissue}1}$) and the second representing a 5 mm thick layer above the inclusion ($\Delta_{\text{tissue}2}$). The arrows illustrate the path of a photon through the tissue, toward the dyed phantom portion, and back to the surface. In the same way as Fig. 6(e), Fig. 7(b) shows TOF as a function of depth, for different IL concentrations. In this case too, a linear relationship was found between TOF and inclusion depth for each IL concentration. These data were corrected for the medium’s optical properties using only SFDI-derived optical property measurements.

### 3.4 Depth Correction

**Fig. 8:**
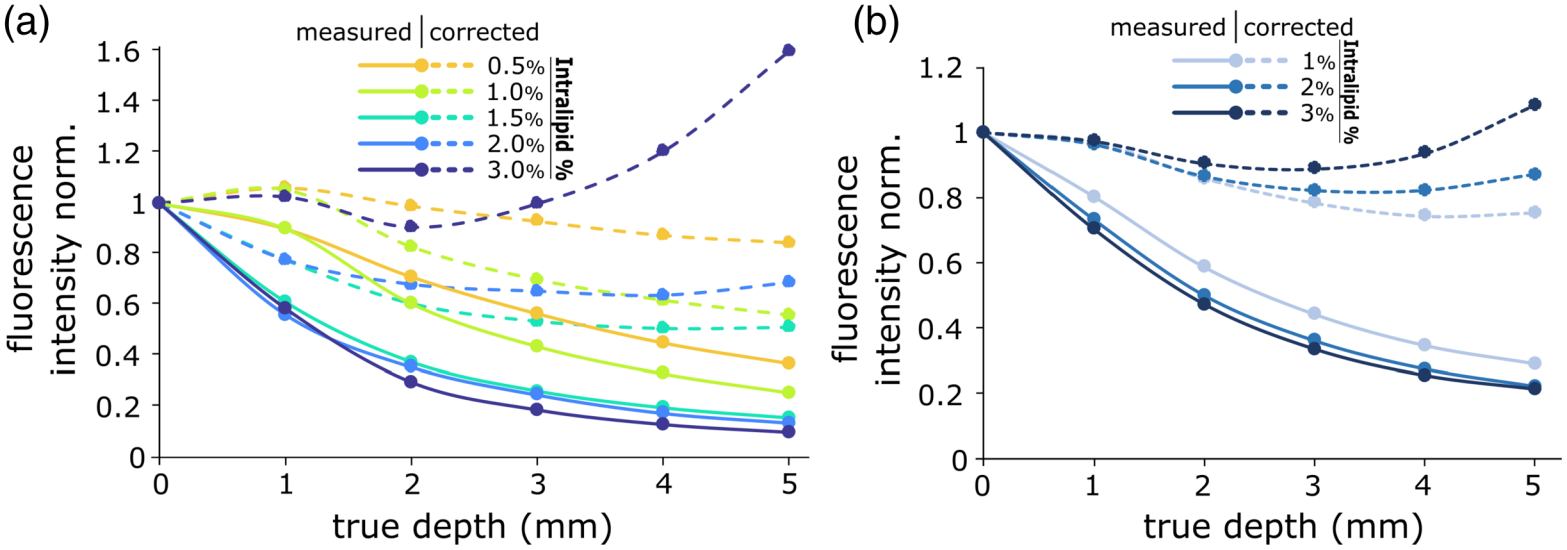
Surface fluorescence intensity at varying inclusion depths (full lines) and corrected intensity representing the luminance at depth (dotted lines) for varying IL concentrations for inclusions embedded in (a) IL aqueous solution and (b) gelatin/blood/IL mixture.

Figure 8 shows the result of surface luminance correction for depth attenuation in both the liquid phantom experiment [Fig. 8(a)] and the porcine gelatin slab phantom [Fig. 8(b)]. In both graphs, fluorescent intensity was measured in terms of photon counts at the phantom surface for varying inclusion depths. These intensities were then normalized to the max intensity for each IL concentration and plotted as solid lines. These data were then corrected using Eq. (10) and plotted as the dotted lines in both graphs. Figure 8(b) demonstrates more consistent intensities as the inclusion depth increases, as a result of depth attenuation correction.

### 3.5 Plastic Tube in Gelatin Phantom

**Fig. 9:**
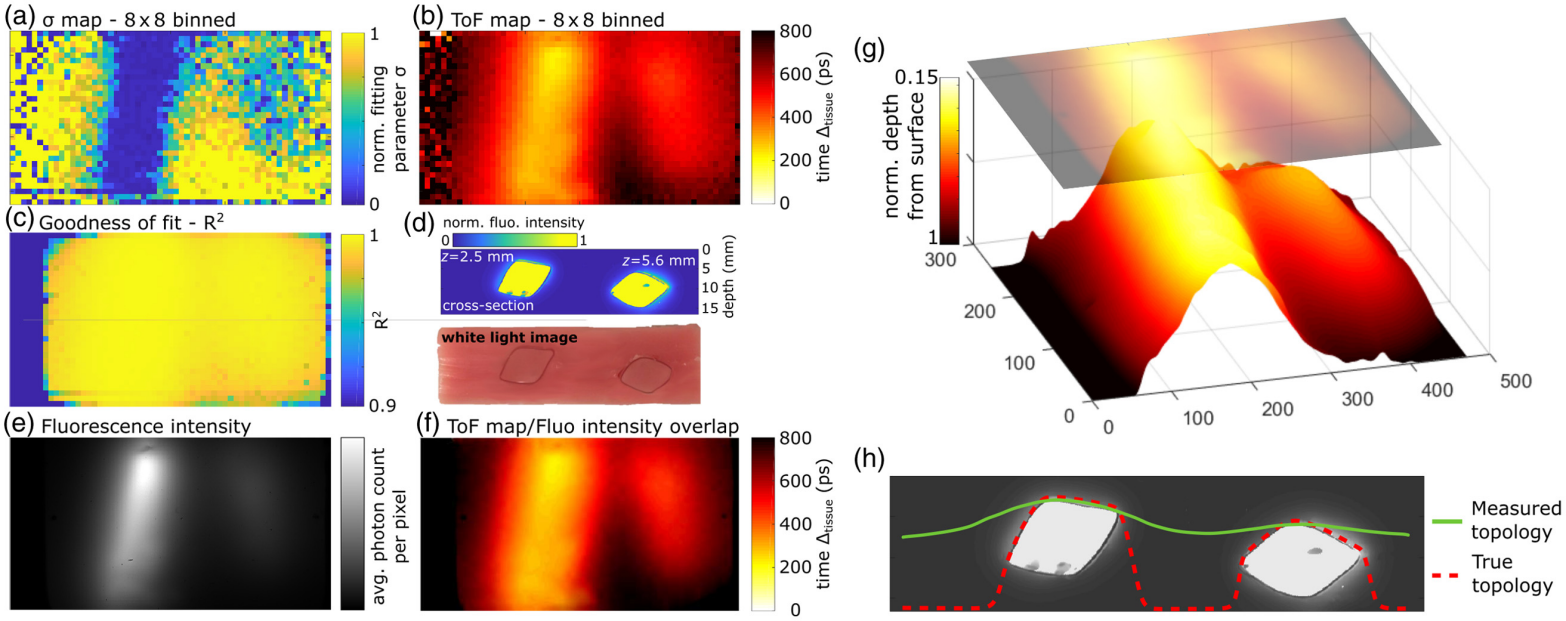
Fluorescent inclusions embedded in the tissue-like gelatine phantom with cylindrical inclusions. (a) Map of Gaussian fitting parameter σ giving a qualitative idea of how much signal coming from two fluorescent tubes gets blurred by the medium. (b) Map of fitting coefficient $\Delta_{\mathrm{tissue}}$, which is a surrogate measurement of TOF. (c) Goodness-of-fit for each 8×8 binned pixel expressed as $R^{2}$. (d) White light image and corresponding fluorescence image of the gelatine phantom cross section. (e) Averaged fluorescence intensity image with histogram equalization for visibility. (f) Overlay of the 8×8 binned TOF map on the equalized intensity picture, which provides background noise cancellation. (g) Topological representation of overlay from (f). (h) Final measured topology compared to true topology overlayed on the phantom cross section.

The last experiment was intended to realistically simulate a fluorescent inclusion in tissue to test the depth calibration developed in the previous section. For this purpose, two fluorescent cylinders were randomly inserted at different depths into a tissue-like phantom composed of porcine gelatin, blood, and IL (see Sec. 2.2.2). The results from the TOF imaging of this phantom are shown in Fig. 9. Figure 9(d) shows a cross section of the phantom. On the bottom is a white light image of the phantom cut in half after imaging, and on the top is a fluorescence image of the same cross section, captured with the Odyssey CLx imaging system. (The white light image does not correspond to the cross-section imaged in this experiment and is only shown for illustration purposes.) Although air gaps can be seen between the inclusion and the surrounding bulk material in the cross-sectional image [Fig. 9(d)], these gaps are a result of cutting the phantom for the representative white light image. Importantly, these gaps were not present during the TOF imaging. Figures 9(a)–9(c) show the results of the measured TPs fitting parameter σ [Fig. 9(a)] and $\Delta_{\text{tissue}}$ [Fig. 9(b)] using 8×8  pixel binning. In this case, the 8×8  pixel binning reduced noise and increased contrast. The SNRs for pixels corresponding to the shallowest and deepest inclusions were ∼10 and ∼2, respectively, in the case of single pixels, and ∼74 and ∼19 when employing the 8×8  pixel binning strategy. The Gaussian fitting parameter σ is a good reporter of the inclusion x−y position in the target plane [Fig. 9(a)]. The inclusion closer to the surface shows up distinctly while the deeper inclusion is visible but less pronounced. Figure 9(b) shows the TOF map corresponding to the fitting factor $\Delta_{\text{tissue}}$. It appears that $\Delta_{\text{tissue}}$ is a good reporter of TOF and shows inclusion depth in more detail. The time scale used for the color bar represents a time relative to the first returning light pulse. Thus the area corresponding to the shortest distance between the inclusion and the phantom surface corresponds to $\Delta_{\text{tisue}}=0$. On the left and right side of the TOF images, the areas showing high noise correspond to the limit of the phantom bulk. Since these areas do not exhibit fluorescence, the fitting model provides inconsistent results as shown on the pixel goodness-of-fit map [Fig. 9(c)]. To overcome this problem, the 8×8 binned TOF image was smoothed using a spatial Gaussian filter [Fig. 9(e)] and overlayed on top of the fluorescence intensity image [Fig. 9(f)]. The goodness-of-fit consistently exhibited values of 0.99±0.03 for pixels corresponding to phantom areas, with these values representing the mean and standard deviation of the $R^{2}$ values, respectively. The resulting image [Fig. 9(f)] shows the final TOF image free of background noise. A 3D representation of the normalized depth is shown in Fig. 9(g), where TOF has been translated to a relative depth from the phantom surface. Figure 9(h) shows the topography obtained from the TOF measurement and corrected using the slab phantom calibration described in Sec. 3.3.

### 3.6 Tumor Resections

**Fig. 10:**
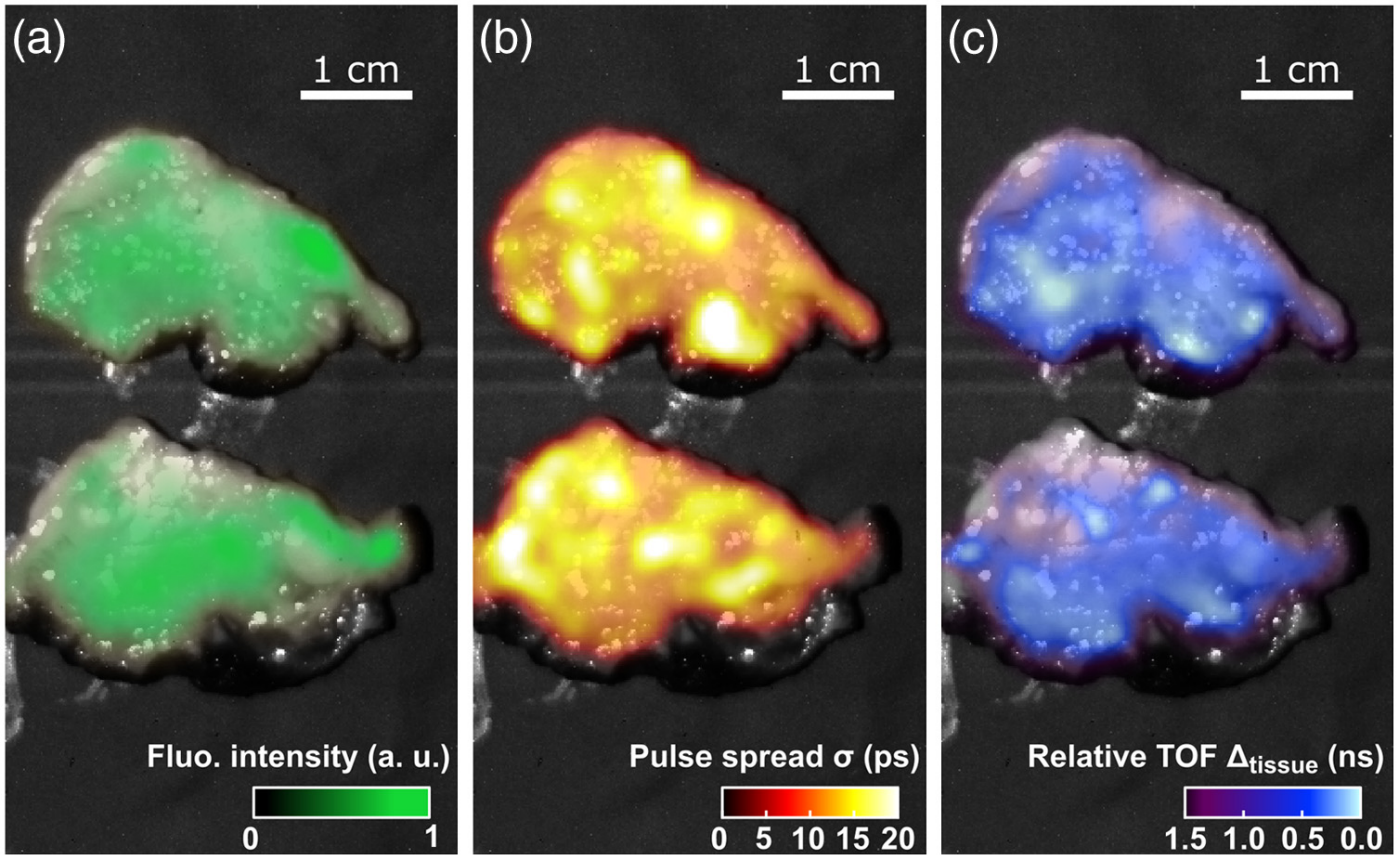
(a) Fluorescent signal from two representative breadloaf slices from two freshly resected head and neck tumors expressed as SNR from an 8×8 binned pixel image, overlayed on top of the reflectance image. (b) Relative fluorescence TOF expressed as $\Delta_{\text{tissue}}$. (c) Map of the fitting parameter σ representing the light pulse time-spread.

Figure 10 shows the result of two labeled specimens from two freshly resected head and neck tumors (details in Sec. 2.2.3). Figure 10(a) shows fluorescence intensity overlayed on a reflectance image of the two tumor slices. The hot spots suggest inhomogeneous distribution of the imaging agent. Figure 10(b) shows the fluorescence TOF map expressed as the fitting factor $\Delta_{\text{tissue}}$. The bright spots observed in the background reflectance image are due to specular reflections. (Cross-polarization filtering was not implemented for this experiment.) Figure 10(c) shows the fitting factor σ representing the excitation pulse’s time spread.

## 4 Discussion

### 4.1 Data Processing

Fluorescence TOF depth sensing using a large-format SPAD camera involved multiple technical aspects, each of which is discussed in the following sections.

#### 4.1.1 SPAD camera settings

In this work, the camera settings were kept constant throughout each experiment. The exposure time per binary frame was set at 4  μs to prevent pile-up effect^35^ when imaging bright objects. Indeed, the high concentration of fluorophores in the experiments depicted in Figs. 5 and 9 produced strong signals, so the exposure time was limited to remain in the linear range of the sensor [as shown in Fig. 2(c)]. Since fluorescence TPs were corrected based on corresponding reflectance TPs, both had to be acquired using the same settings. In the experiments shown in Figs. 7 and 10, reflectance showed an intensity two orders of magnitude higher than fluorescence, and the average pixel count for 3% IL in porcine gelatin at 5 mm depth was as low as 0.4 photons per pixel per exposure. One possible solution to this problem would be to increase exposure time and use ND filters to reduce reflectance intensity. However, this would complicate the data acquisition process, which had to be kept simple for nonexperts to acquire data in a short amount of time. Additional engineering is required to achieve full automation in handling intensity variations, ensuring a more comprehensive and refined prototype of the system.

#### 4.1.2 Time profile comparison

One of the main reasons to acquire reflectance TPs was to correct fluorescence TPs for the sample’s geometry and changes between measurements. Reflectance TPs are mostly affected by large object surface-to-sensor distances as it takes a resolution of ∼17  ps to resolve 2.5 mm based on the speed of light in air. Reflectance data are thus only useful to measure significant changes in surface geometry given our resolution of 17.8 ps. Here target surface variations were in the millimeter range, and consequently, the reflectance TPs were assumed to be constant for each setup geometry. When the setup was changed in between two acquisitions, reflectance TPs were used to correct fluorescent TPs.

#### 4.1.3 Time profile fitting

Fitting TPs was essential to quantify time-resolved information. Indeed, as a turbid medium’s absorbance and/or scattering increases, or the overall distance traveled through the medium increases, the TOF signal weakens. Most raw data collected in this study exhibited low SNR. Consequently, finding appropriate models to fit the TPs enabled the recovery of information despite the high noise level. The fitting model used in this study was simplistic but was found to be efficient. However, it has some limitations when resolving small details or variations, as seen in the characterization of optical properties using reflectance data. TP fitting on all pixels using the entire, unbinned sensor took ∼4  h. However, by binning the pixels 8×8, this time is reduced to just a few minutes per image.

### 4.2 Liquid Phantoms

The first part of this study aimed to determine if TOF as measured by the system was a good reporter of fluorescent inclusion depth in turbid media. The first set of experiments focused on measuring fluorescent TP from a phantom where the inclusion depth was controlled precisely. The liquid phantom consisted of IL in aqueous solution at varied concentrations. This phantom was a simplified case since its absorption was close to 0. That allowed for increased signal and improved TOF sensing at depth.

Even though inclusion depth was controlled precisely, Fig. 5(e) shows that the five linear plots do not converge at a depth of 0, which corresponds to the case where the inclusion was not surrounded by any liquid. The main reason for this discrepancy is that the first addition of surrounding liquid was not precise due to superficial tension at the surface of the sample. As a result, the depths shown in Fig. 5(e) are not completely accurate. However, once the superficial tension was broken, a controlled thickness of solution could be added, making the change in depth precise. For this reason, in Fig. 5(e), linearity is conserved from 1 to 5 mm, but a slight offset is observed between 0 and 1 mm. As a result, the data for depths between 0 and 1 mm were not included in the analysis. The coefficient $c_{\mathrm{fluo}}$ from Eqs. (10) and (11) is in theory constant and only depends on the setup geometry.

Another element that was not considered in this set of experiments is the 1 mm thickness of the plastic cuvette’s wall. Due to the speed of light in a transparent medium, it is not possible, given the temporal resolution available, to resolve such thin layers. Therefore, it was assumed that the plastic walls had a negligible influence on the time-resolved data.

The goal of this first experiment was to highlight the relationship between measured TOF and inclusion depth. It was shown that this relationship is linear in the observed depth range (1 to 5 mm). It also showed that linearity was maintained at various levels of optical scattering. The higher the scattering coefficient is, the faster TOF increases with depth is. This is shown by the red curve in Fig. 5(e), which reports the slope $k_{\mathrm{fluo}}$ as a function of IL concentration. One final take away from this first set of experiments was that the system’s ability to resolve sub-millimeter depth was due to the optical properties of the target medium (i.e., high scattering). The depth sensitivity achieved in this set of experiments would not have been possible otherwise, even considering the high refractive index of the medium.

### 4.3 Depth Calibration

Depth calibration can be achieved by quantifying changes induced by varied optical properties, as described by Eqs. (10) and (11). Two methods were investigated for this purpose: first, reflectance-based calibration using direct time-resolved measurements from the SPAD camera; and second, using an established external modality (i.e., SFDI). Both calibration models were developed empirically by observing the relationship between different parameters. It should be noted that while these methods provide quantification of bulk optical properties in surface tissues, they do not possess the capacity to evaluate these parameters in terms of depth. Consequently, the general approach presented in this study operates under the assumption that variations in optical properties are insignificant along the z axis.

#### 4.3.1 Reflectance data

Time-resolved imaging of reflectance can be informative of medium optical properties depending on the time resolution available. When propagating through a turbid medium, light intensity decays exponentially with depth.^33^^,^^36^^,^^37^ As a result, light resulting from diffuse reflectance is heavily surface weighted. Unlike fluorescence that results from longer, scattered paths, diffuse reflectance only carries information resulting from shorter light–matter interaction and is therefore less impacted by the optical properties of the medium.

Thus reflectance TPs were first used as a time-resolved measure of medium optical properties, independently from the fluorescent inclusion depth. Figure 6(a) shows that reflectance TPs only vary slightly at the end of the rising edge. For this reason, in this case only, reflectance TPs were matched on their falling edges. The values of these five different curves at 2% of the normalized pulse exhibited a linear relation with IL concentration. Figure 6(c) shows this relation for different IL%, used for data calibration. Data were extrapolated from this curve for 0.5% and 1.5% Intralipid solution in the case of the first experiment. The same method was used later to measure tissue optical properties from tissue-like gelatin phantoms to correct data from the gelatin slabs experiment. Reflectance TPs showed close to no sensitivity to optical properties in that case. We assume that this is because these phantoms had a higher absorption coefficient due to the presence of blood, which led to more absorption of the signal traveling through the medium’s superficial layer.

To conclude, using reflectance data as a fast measure of medium’s optical properties was not suitable for realistic measurement conditions. Our results were not conclusive for simplified phantoms. Higher time-resolution systems and refined fitting models could enhance the efficacy of this method. Alternatively, SFDI presents a viable option for achieving rapid and noncontact measurements of tissue optical properties.

#### 4.3.2 Spatial frequency domain imaging

SFDI is a robust, well-documented method to recover tissue optical properties. It allows for precise, pixel-level medium optical properties characterization. It has the advantage to be able to recover both $\mu_{a}$ and $\mu_{s}^{\prime }$, which is not the case of the reflectance TP method. Therefore, $\mu_{\mathrm{eff}}$ can be recovered to correct for depth attenuation as explained in Sec. 2.4.3. The commercial SFDI system measured optical properties at 8 wavelengths. For our system calibration and data correction, optical properties quantified at 659 nm were used as a compromise between the fluorophore excitation wavelength (635 nm) and the peak emission wavelength (680 nm).

### 4.4 Gelatin Slabs Phantom

The second experiment involved a phantom with more realistic parameters compared to the first liquid phantom experiment. Porcine gelatin, bovine blood, and IL were used to fabricate a tissue-like phantom. The fluorescent inclusions were dyed with IRDye 680LT at a concentration of 50 nM (versus 10  μM for the liquid phantom). The 1 mm gelatin slabs offered precise control of the inclusion depth from 0 to 5 mm depth. Since the dye concentration was relatively low, the system was upgraded with a larger aperture lens and a shorter working distance. Results from Fig. 7(b) show that in the same way as in Fig. 5(e), there is a linear relationship between TOF and fluorescent inclusion depth. Data from this experiment were noisier than the data from the previous experiment. TOF data at 5 mm were not shown here, because signal reached the noise level near a depth of 4 mm due to the medium’s relatively high absorbance.

This experiment showed the limit of the SPAD LiDAR system. Even though TOF reconstruction is theoretically insensitive to fluorescence intensity, a low SNR is correlated with TP fitting inaccuracies. Averaging signal over a larger pixel area allowed for better results but at the cost of reduced spatial resolution.

### 4.5 Plastic Tube in GELATIN Phantom

The third experiment involved a fluorescent tube embedded in gelatin. The phantom was fabricated in a way that the inclusion position in space was not precisely known. Therefore, this experiment more closely emulated a real-life scenario where the inverse problem had to be solved using the calibration based on the gelatin slab phantom. Upon analyzing the results depicted in Figs. 5(a) and 5(b), it appeared that the spread of TPs did not change with inclusion depth or IL concentration. However, a closer examination in Fig. 9(a) revealed that the pulse width, denoted as σ, was indeed affected by depth. The result was noisy, and the deeper inclusion was only slightly discernable in Fig. 9(a). TOF, expressed as $\Delta_{\text{tissue}}$, on the other hand, seemed to be a more effective reporter of the inclusion position. When comparing Figs. 9(g) and 9(h), the relative distance between objects was conserved. For both fitting parameters, 8×8 binned pixel images were used, as it improved contrast, particularly in the case of σ. For $\Delta_{\text{tissue}}$, pixel binning did not have a significant impact on the result. The outgrowth seen at the bottom right of the shallowest tube was due to the transparent glue used to seal the tube. As it is transparent, the measured TOF is reduced as it acts like a waveguide; it does not represent a fluorescent inclusion.

To differentiate background noise from actual signal, the TOF map was Gaussian filtered and overlayed on the fluorescence intensity image. To ensure accuracy, only TOF measurements from regions expressing fluorescence were retained. However, this method has the disadvantage of discarding information in regions with low fluorescence intensity, such as the bottom right side of Fig. 9(f). This experiment aimed to simulate real condition measures where the spatial positions of inclusions were unknown, the goal being to locate them in space.

These combined results helped to verify that the methodology employed in this study would be robust for recovering the spatial positions of inclusions within tissue. However, the depth measurement capability has limits in cases where inclusions are located close to each other, due to scattering. Further deconvolution to account for high scattering is needed to obtain more accurate depth measurements.

### 4.6 Tumor Resections

Finally, we were able to acquire SPAD LiDAR images of representative tumor slices from two freshly resected head and neck tumors, carried out as part of a larger ongoing study. In this case, signal levels were low, because the tumor was labeled with ABY-029 at lower (near microdose-level) concentration (SNR of ∼1.4 for single pixels and SNR of ∼3 for 8×8 binned pixels). Furthermore, ABY-029 is an Affibody molecule labeled with IRDye 800CW, whose excitation and collection spectra did not align optimally with the parameters used in our system. Nevertheless, it was an excellent opportunity to image human tissue that had just been resected, albeit without being able to ultimately compare to the ground truth of depth from which the fluorescence came.

The σ map [Fig. 10(c)] did not appear to be correlated with the TOF map. This could be due to both the low SNR of the dataset, providing poor fitting of fine parameters, and/or inhomogeneities in the tissue optical properties.

The tumor tissue samples provided a first assessment of how the SPAD LiDAR TOF approach performs when imaging biological samples with irregular surface profiles. While the true depths remain unknown, the system demonstrated consistent results in terms of TOF range and tumor distribution across the resection samples.

To address the challenges arising from the dataset’s low fluorescence intensities and pronounced noise levels, we implemented an 8 × 8 binning technique for the sensor. Although our initial aim in employing a large-format SPAD was to enhance spatial resolution, our findings revealed that pixel binning proved effective in overcoming these limitations and restoring valuable SNR. Through the consolidation of pixels via binning, we still achieved a sufficient spatial resolution, enabling the visualization of intricate biological details with a fine resolution down to 1 mm in scale—particularly evident in the context of tumor resection imaging.

### 4.7 System Improvement and Opening

The use of a high-resolution, time-resolved SPAD camera as a LiDAR system was overall a success. Absolute time-resolved reading from the system was not quantitatively informative of true depth. To achieve true depth measurements, the system needs to be calibrated for the target medium’s specific distributions of optical properties. To be able to efficiently work this way, a lookup table with calibration corresponding to various combinations of optical properties would be needed. Machine learning could help address this aspect of system improvement.^38^^,^^39^

In any case, the SPAD LiDAR method still requires a way to quantify medium’s optical properties. It was found that using SFDI was a robust way to do so, at the expense of requiring a separate imaging setup and additional processing time. Alternatively, time-resolved, patterned light projection composed of an array of dots could be used to recover medium’s properties.^40^ This would offer a compact method to correct TOF data that leverages the same SPAD sensor.

Finally, increasing excitation power could be a way to significantly enhance signal without any further modification, providing better TOF approximation for low light level situations.

## 5 Conclusion

In this study, we explored the use of a large-format SPAD sensor to localize and quantify the concentration of fluorescent molecules in a scattering medium, such as biological tissue. Several methods had to be incorporated into the approach (i.e., recovery of the tissue optical properties, analytical model fitting of the concentration, and depth parameters). Testing in a series of tissue-like phantoms and eventually in resected tissues allowed for varying levels of validation of this methodology. The system’s high time resolution enabled sub-millimeter depth accuracy, and the sensitivity was sufficient to quantify fluorescence TOF signals down to 5 mm deep into human tissues. In addition to classical molecular imaging techniques, fluorescence LiDAR provides the capability to image depth information over a wide field of view, which when fully integrated into a system, will allow augmented surgical vision below the tissue surface.

## Data Availability

All data and MATLAB routines are available upon request.
